# Identification and Characterization of a Novel Multipotent Sub-Population of Sca-1^+^ Cardiac Progenitor Cells for Myocardial Regeneration

**DOI:** 10.1371/journal.pone.0025265

**Published:** 2011-09-28

**Authors:** Michitaka Takamiya, Khawaja H. Haider, Muhammad Ashraf

**Affiliations:** Department of Pathology, University of Cincinnati, Cincinnati, Ohio, United States of America; Northwestern University, United States of America

## Abstract

**Methods and Results:**

The cardiac stem/progenitor cells from adult mice were seeded at low density in serum-free medium. The colonies thus obtained were expanded separately and assessed for expression of stem cell antigen-1 (Sca-1). Two colonies each with high Sca-1 (CSH1; 95.9%; CSH2; 90.6%) and low Sca-1 (CSL1; 37.1%; CSL2; 17.4%) expressing cells were selected for further studies. Sca-1^+^ cells (98.4%) isolated using Magnetic Cell Sorting System (MACS) from the hearts were used as a control. Although the selected populations were similar in surface marker expression (low in c-kit, CD45, CD34, CD31 and high in CD29), these cells exhibited diverse differentiation potential. Unlike CSH1, CSH2 expressed Nanog, TERT, Bcrp1, Nestin, Musashi1 and Isl-1, and also showed differentiation into osteogenic, chondrogenic, smooth muscle, endothelial and cardiac lineages. MACS sorted cells exhibited similar tendency albeit with relatively weaker differentiation potential. Transplantation of CSH2 cells into infarcted heart showed attenuated infarction size, significantly preserved left ventricular function and anterior wall thickness, and increased capillary density. We also observed direct differentiation of transplanted cells into endothelium and cardiomyocytes.

**Conclusions:**

The cardiac stem/progenitor cells isolated by a combined clonal selection and surface marker approach possessed multiple stem cell features important for cardiac regeneration.

## Introduction

The pioneering work of Anversa *et al.* has provided an intriguing evidence of increased mitotic activity in the cardiomyocytes in patients with myocardial infarction (MI) besides the existence of resident population of cardiac stem/progenitor cells [1]. These results are supported by other research groups to show that resident stem/progenitor cells have the ability to differentiate into all cell types required for myocardial regeneration including cardiomyocytes, smooth muscle cells and endothelial cells [2]. Despite their immense differentiation potential, use of cardiac stem/progenitor cells is limited due to lack of markers for selection of a specific stem cell population [3]. Stem cell antigen-1^+^ (Sca-1^+^) [3], [4], Islet-1^+^ (Isl-1^+^) [5], c-kit^+1^, side population (SP) cells [6], [7] and cardiosphere-derived cells [8] have been extracted from heart tissue. Nevertheless, there is no distinct relationship between these reported cardiac stem/progenitor cell populations due to difference in isolation and characterization methods. Moreover, it remains to be determined whether these individual stem/progenitor cell populations represent different intermediate developmental stages of the same stem/progenitor cells or these are independent cell lineages which have originated from different stem/progenitor cell populations. Similarly, their relationship with the cardiac precursor cells observed during embryonic development has not been established [9], [10].

In this study, we isolated several sub-populations purified from adult mouse heart based on clonogenicity and surface marker expression and investigated the important features of cardiac stem/progenitor cell population for cardiac differentiation.

## Materials and Methods

### Ethics statement and the Animals used

C57BL6 mice (Harlan) were used for cell isolation and cell transplantation models. All animal experiments conformed to the Guidelines for Care and Use of Laboratory Animals published by the US National Institutes of Health (NIH Publication No. 85-23, revised 1985) and all protocols of animal experiments were approved by the Institutional Animal Care and Use Committee, University of Cincinnati, Protocol #06-03-13-03.

### Cell culture

The whole hearts extracted from male C57BL6 mice (12 weeks old) were perfused and washed several times with ice-cold PBS to remove the blood cells. Aorta, pulmonary artery, and pericardium were removed carefully. The hearts were minced and digested for 20-minutes at 37°C with 0.1% type-II collagenase (Invitrogen) and 0.01% DNAse-I (Worthington Biochemical Corporation). The cells were passed through 40 µm filter, fractionated with 70%Percoll (Fluka) and cultured in maintenance medium containing serum-free DMEM/F12 (Invitrogen) supplemented with B27 (Invitrogen), 20 ng/ml epidermal growth factor (EGF; Sigma), and 40 ng/ml basic fibroblast growth factor (bFGF; PeproTech). The cells were collected 1-week later and re-seeded on new culture dishes with serum-free maintenance medium at a low density (100cells/cm^2^) to initiate colony formation. Sixteen days later, the colonies thus obtained were transferred into 24-well dish and cultured individually in expansion medium containing DMEM/F12 (Invitrogen) supplemented with 2%FBS, B27-Supplement (Invitrogen), 20 ng/ml EGF (Sigma), 40 ng/ml bFGF (PeproTech), and 10 ng/ml leukemia inhibitory factor (LIF; Chemicon). All colony-derived cells were re-seeded on new dishes after they reached 90–100% confluence and were maintained with expansion medium.

Sca-1^+^ cell-enriched population was also purified from the heart-derived cells with Magnetic Cell Sorting system anti-Sca-1 MicroBead Kit (Miltenyi Biotec) per instructions of the manufacturer and maintained in expansion medium similar to other colony-derived cell populations.

### Flow cytometry

Flow cytometery of the purified cell populations was carried out as described in Text S1.

### Growth function assay

Each cell population was seeded at a density of 1×10^4^ cells/cm^2^ in 1 ml expansion medium on 24-well plate and cultured at 37°C in humid air with 5% CO_2_. Cell number was counted on days 1,3,5, and 7 after initial seeding. The doubling time of each cell population was calculated with the cell numbers on day 1 and 3 [11].

### RNA extraction and RT-PCR

Total RNA was extracted from different treatment cell populations with RNeasy Mini Kit (Qiagen) and cDNA was generated with Omniscript-RT Kit (Qiagen). Each PCR reaction was performed with target gene specific primers. We also performed real-time PCR to determine the expression of VEGF gene under anoxic conditions using SYBR-Green Master Mix (BIO-RAD) in a BIO-RAD-iQ5 optical module. The mRNA level was standardized to inner control (GAPDH) and expressed as fold-increase versus normoxia condition.

### Sphere formation assay

Sphere formation assay was performed as described in Text S1.

### Differentiation assay

These cells were cultured in specific differentiation media for smooth muscle, endothelial cells, cardiomyocyte, osteogenic, chondrogenic, and adipogenic differentiation as detailed in Text S1 and detected by specific staining as described earlier [12].

### Myocardial infarction model and cell transplantation

Experimental MI was developed in young female C57BL6 mice (12-weeks old; 20–25 g body weight) by left anterior descending (LAD) coronary artery ligation [13]. Ten minutes later, 20 µl basal DMEM/F12 medium without cells (control group) or containing 2×10^5^cells (Cell Injection group) were injected into infarction and border zones as described in Text S1.

### Echocardiography

The mouse heart function was assessed by transthoracic echocardiography before surgery (baseline), 1 and 4-weeks after surgery using 7–15 MHz probe on iE33 Echocardiography system (Philips) (Please see Text S1).

### Morphological analysis

The hearts were prepared for measurement of infarction size, anterior wall thickness, and percent fibrosis as described earlier [14] and detailed in Text S1.

### Capillary density analysis

Capillary density was determined as described earlier [15] and detailed in Text S1.

### Fate tracking of the transplanted cells and histological studies

The cells were labeled with red fluorescent nanocrystals, Qtracker®625 Cell Labeling Kit (Invitrogen) before transplantation according to manufacturer's instructions. The hearts were harvested 1, 2 and 4-weeks after cell transplantation and perfused with ice-cold PBS. The extracted hearts were cut transversely and embedded into OCT compound (Sakura Finetek) and frozen with liquid nitrogen. Semi-thin cryosections (10 µm) were fixed in acetone at −20°C for 10 minutes and blocked with CAS BLOCK™ (Invitrogen). Sections were incubated with anti-von Willebrand Factor (Chemicon, dilution = 1∶100) or anti-cardiac troponin-I (cTn-I) antibody (Santa Cruz; 1∶100) for 1-hour at 37°C and subsequently incubated with secondary antibody conjugated to Alexa Fluor-488 (Invitrogen) for 1-hour at 37°C. Nuclei were stained with DAPI.

### Co-culture assay for myogenic differentiation

Neonatal rat cardiomyocytes were isolated from 1–3days old rat pups using Neonatal Cardiomyocyte Isolation System (Worthington Biochemical Corporation). For co-culture studies, cardiac cells were stained with Qtracker®625 Cell Labeling Kit (Invitrogen) and mixed with freshly isolated cardiomyocytes (1∶4) and seeded on the fibronectin-coated chamber slides at a density of 2.5×10^4^/cm^2^. For co-culture of the purified cardiac stem/progenitor cells with fixed neonatal cardiomyocytes, freshly isolated cardiomyocytes were seeded on fibronectin-coated chamber slides at a density of 2×10^4^/cm^2^. After 24-hours culture, cardiomyocytes were fixed with 0.5%PFA for 15 minutes at room temperature and washed with PBS [5]. Qtracker-labeled purified cardiac stem/progenitor cells were seeded on fixed cardiomyocytes at a density of 5×10^3^/cm^2^. Co-culture of the purified cardiac stem/progenitor cells with live or fixed-cardiomyocytes were maintained for 6-days and, cells were fixed with 4%PFA and immunostained for anti α-sarcomeric actinin.

### Statistical analysis

All data were expressed as mean±SD. Comparison between two mean values was calculated by an unpaired Student 2-tailed *t*-test. Comparison between multiple groups was performed by one-way ANOVA with post hoc analysis. A value of *p*<0.05 was considered statistically significant.

## Results

### Characterization of clonogenic cell populations

**Figure 1:**
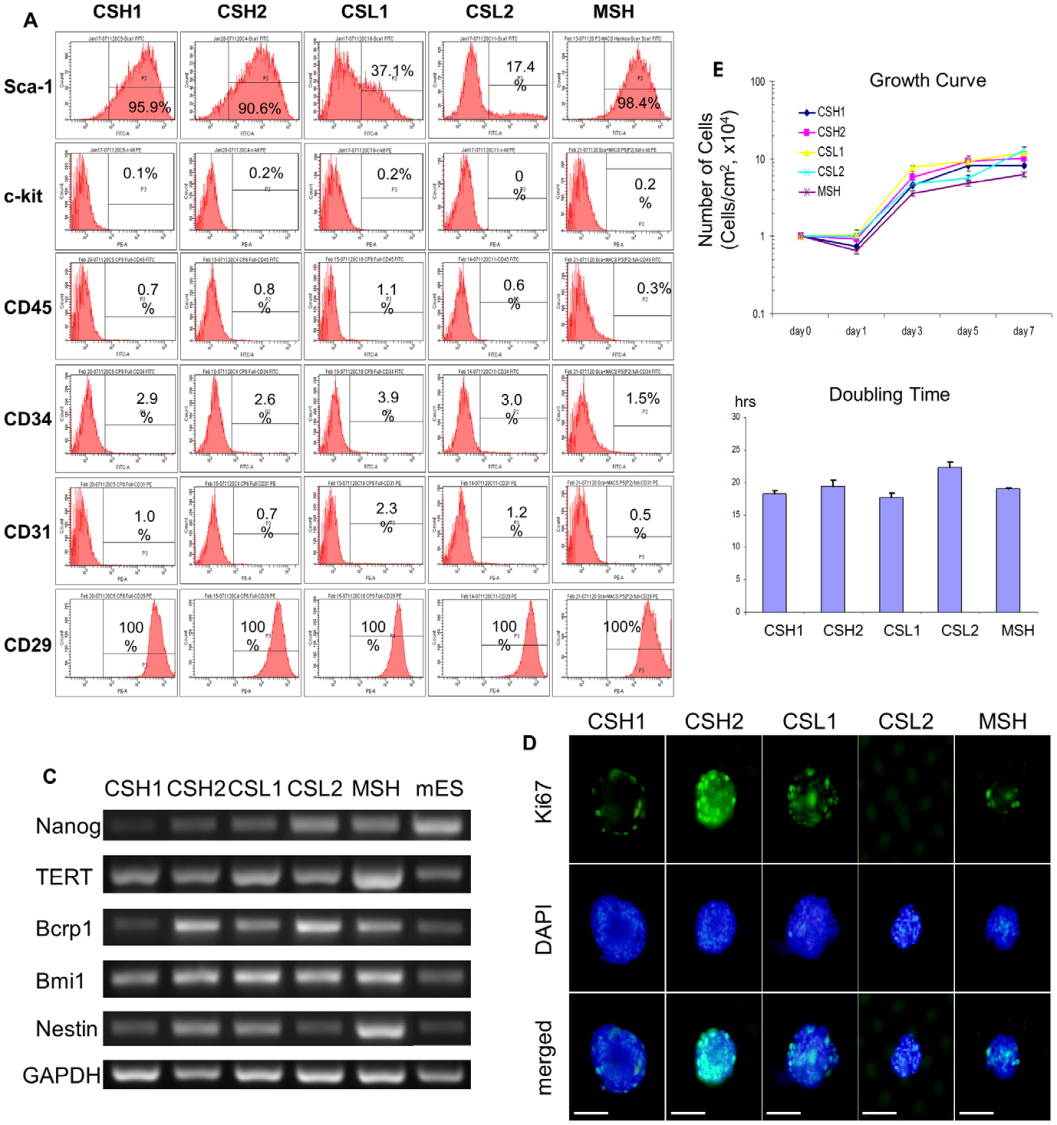
Characteristics and genetic background of colony-derived cell populations obtained from adult mouse heart. A, Surface marker expression analysis by flow cytometry. All selected cell populations were analyzed for expression of Sca-1, c-kit, CD45, CD34, CD31, and CD29. B, Left, Growth curve of individual cell populations in expansion medium. Right, calculated doubling time of individual cell populations (*p<0.01 *vs* all other cell populations). Abbreviations: CSH, colony-derived Sca-1 high expression group; CSL, colony-derived Sca-1 low expression group; MSH, magnetic beads sorted Sca-1 high expression group. C, Expression of stem cell-related genes analyzed by RT-PCR. RNA extracted from mouse embryonic stem (mES) cells was used as positive control. D, Sphere-like cell cluster formed on ultra-low attaching dish was stained with Ki67 (green) and DAPI (blue). Bar = 100 µm.

The colony-derived cell populations were analyzed for Sca-1 and c-kit expression (Figure 1A). Sca-1 expression varied from 95.9% to 17.4% whereas c-kit expression was barely detected. The cell isolation procedure was successfully repeated three times to study the reproducibility of the procedure. We selected two high Sca-1 expressing populations (CSH1 and CSH2), and two low Sca-1 expressing populations (CSL1 and CSL2). Non-colony-derived Sca-1^+^ cells selected with MACS (MSH) were used as controls. These cells rarely expressed CD45, a known hematopoietic lineage marker. Besides, these cells occasionally expressed hematopoietic progenitor and endothelial cell markers CD34 and CD31 [16], [17] whereas frequently expressed mesenchymal stem cell marker CD44. Although all of colony-derived cells except CSL2 showed similar short doubling-time to embryonic stem cells, only CSL2 showed significantly longer doubling-time compared to other cells (Figure 1B). Non-colony-derived Sca-1 cells, MSH, also showed similar doubling-time. These results were consistent with the previous reports [18].

### Molecular characteristics and clonogenicity of cardiac cell populations

Gene expression profile showed that these cell populations were positive for pluripotent stem cell markers Nanog, Telomerase reverse transcriptase (TERT, which is usually not detectable in cardiac fibroblasts) [19], and Bcrp1/ABCG2 (which play central role to form stem cell-enriched “side population”) [6] (Figure 1C). Bmi1 gene, a determinant of self-renewal of hematopoietic and neural stem cell was also detected [20]. These pluripotency markers were detectable in all the cell populations, however, the expression of Nanog and Bcrp1 was obviously weaker in CSH1 cells. These data suggested that these cells had different genetic characteristics compared to the cardiac fibroblasts or endothelial cells.

Sphere formation assay ascertained the self-renewal capacity of the isolated cells [8] and showed sphere-like cell aggregation. However, sphere formation with homogeneous positive staining for Ki67 was detected in CSH2 cells only (Figure 1D).

### Differentiation potential of the cells into cardiac cell lineage

We assessed the differentiation potential of these cells into smooth muscle, endothelial, and cardiac phenotypes. After stimulation with platelet derived growth factor-BB (PDGF-BB), these cells showed significant increase in calponin positive cell count (Figure S1 A–B) which was confirmed by RT-PCR (Figure S1 C). Two weeks after induction for endothelial cell differentiation, vWF^+^ cells were detected only in CSH2, CSL2, and MSH populations (Figure S2A) whereas we did not observe expression of endothelial specific markers in any of the cell populations before induction of differentiation (Figure S2B–C). However, a statistically significant increase in vWF^+^ cells was only observed in CSH2 cells (Figure S2). The expression of endothelial cell specific genes, CD31, VE-cadherin, and vWF was confirmed by RT-PCR in CSH2, CSL2, and MSH populations.

### Cardiomyogenic differentiation

**Figure 2:**
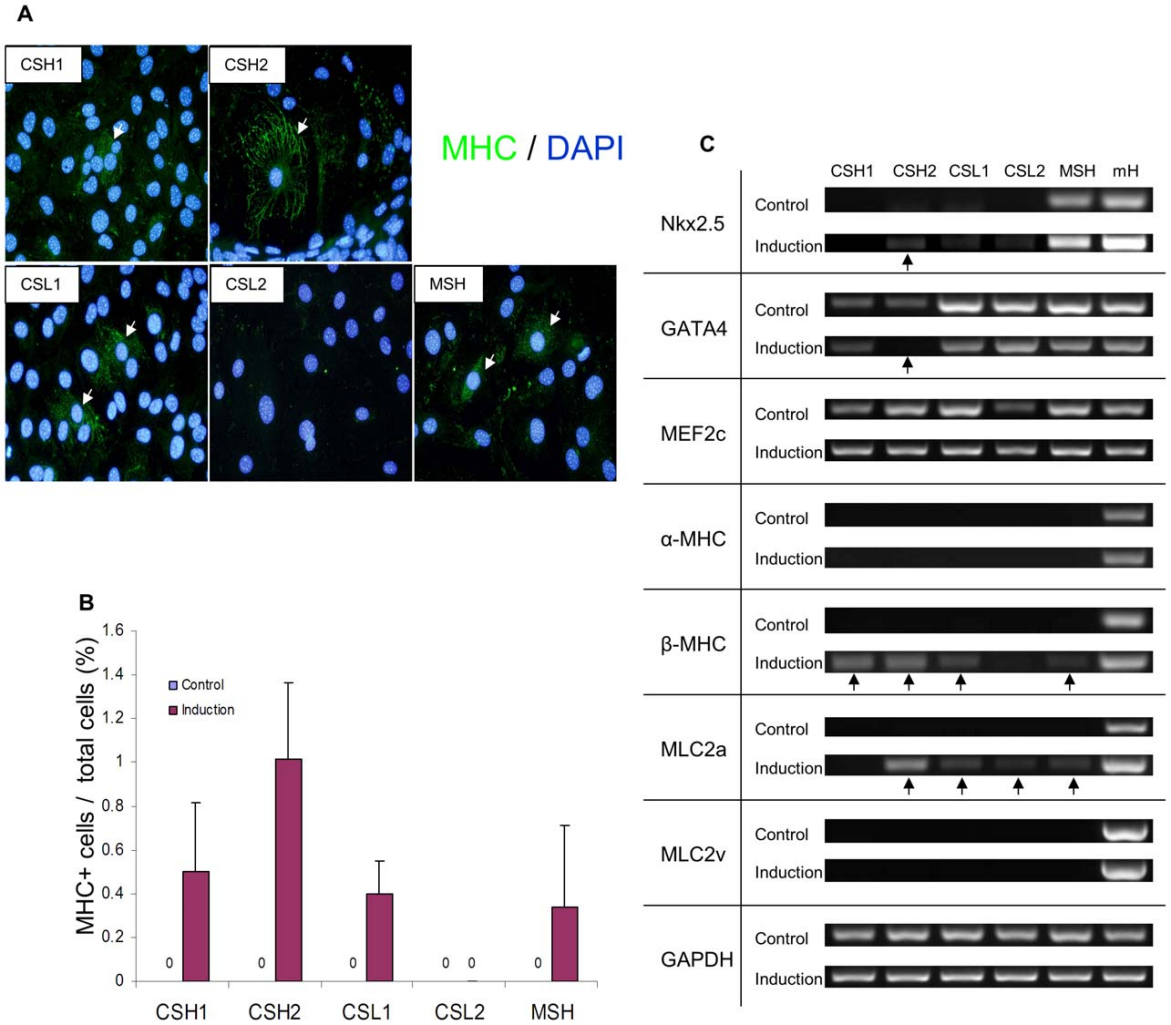
Differentiation potential of isolated Sca-1 high or low expressing cell populations. A, All cell populations were stained for cardiomyocyte specific marker cardiac MHC (green) and DAPI (blue) after induction. White arrow indicated the cells stained positively for cardiac MHC. Magnification = ×400. B, Cardiac MHC positive cells were calculated before induction (control) and after induction (Induction). The positive rate was presented as ratio of MHC positive cell number to total cell number (*p<0.01 *vs* control; **p<0.05 *vs* control; † *p*<0.05 *vs* CSL1, CLS2, and MSH). C, Expression of cardiomyocyte specific genes analyzed by RT-PCR. RNA extracted from whole adult mouse heart (mH) was used as positive control. Arrow indicated the change of cardiomyocyte specific gene expression after induction.

Quantitative analysis of cardiac MHC^+^ cells revealed highest differentiation potential in CSH2 population (Figure 2A, 2B). At the baseline, the early cardiac marker gene, Nkx2.5 was detected only in MSH population (Figure 2C) whereas GATA4 and MEF2C were detected in all cell populations (Figure 2C). On the contrary, mature cardiomyocyte marker genes, cardiac α-myosin heavy chain (α-MHC), cardiac β-myosin heavy chain (β-MHC), myosin light chain 2a (MLC2a), and myosin light chain-2v (MLC2v) were undetectable in all cell populations. Cardiogenic differentiation was attempted with dexamethasone, DMSO, 5-azacytidine, oxytocine, TGF-β, Wnt1, Wnt5a, BMP2, and FGF4, individually or in combination with two or more agents. Although these agents failed to generate mature beating cardiomyocytes invariably in all the purified cells, stimulation with BMP2 combined with FGF-4 induced cardiac mature marker genes in several cell populations (Figure 2C). Only CSH2 cell population showed multiple cardiac genes (Nkx2.5, β-MHC, and MLC2a) after induction (Figure 2C). MSH population showed only limited cardiomyogenic gene expression (Figure 2C) despite the expression of early cardiogenic marker genes Nkx2.5, GATA4, and MEF2c prior to induction.

### Genetic background and differentiation potential of Sca-1^+^ cells

**Figure 3:**
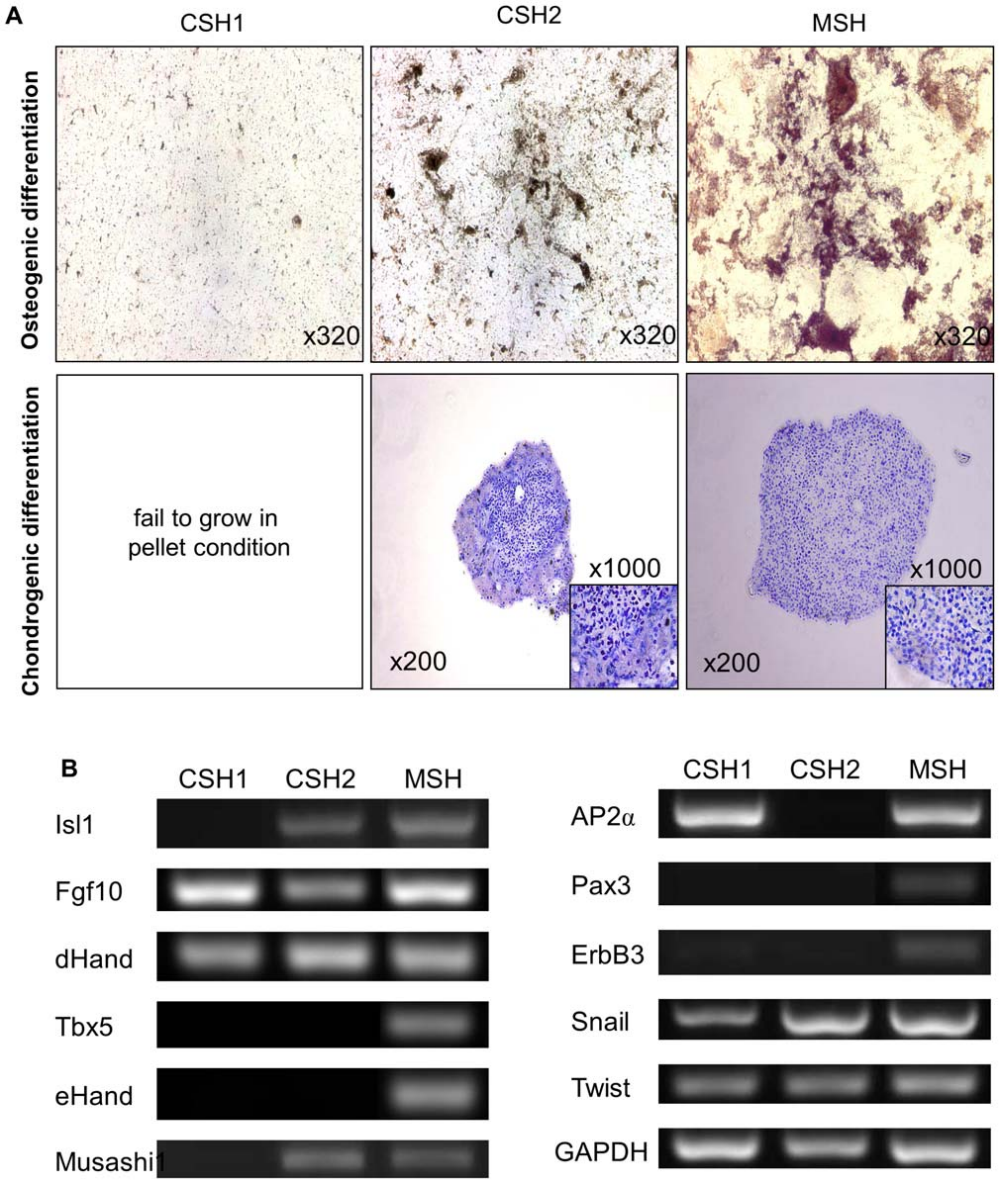
Multiple differentiation potential of isolated colony-derived Sca-1 high expressing population. A, Differentiation assay of Sca-1 high expressing populations into mesenchymal cell lineage. Upper panel shows osteogenic differentiation of Sca-1 high expressing populations with von Kossa staining. Magnification = ×320. Lower panel shows chondrogenic differentiation with Toluidine Blue O staining. Magnification = ×200. Inserts show magnified area from same sample. Magnification = ×1000. B, Gene expression profiles of Sca-1 high expressing cell populations analyzed by RT-PCR.

Sca-1 is commonly used for enrichment of haematopoietic stem cells [21]. Certainly, Sca-1 high expressing population, CSH2, showed obvious and unique stem/progenitor cell characteristics in all analyses. However, Sca-1 high expressing population, CSH1, showed poor potential inspite of similar isolation method, growth function, and high expression of Sca-1 antigen compared to CSH2. Differentiation assay showed that these cells failed to differentiate into adipogenic lineage, whereas CSH2 and MSH showed obvious differentiation potential into osteogenic and chondrogenic lineages (Figure 3A). CSH1 did not differentiate into any of these three different cell lineages.

We also performed gene expression analysis to determine additional difference between Sca-1 high cell populations. The gene expression of LIM homeodomain transcription factor islet-1 (Isl1), the marker of cardiac precursor in the developing as well as postnatal heart [5], was detected in CSH2 and MSH but not in CSH1 cells (Figure 3B). Since Isl1 is the marker of cells from secondary heart field in developing heart [9], we checked the expression of FGF10 and dHand gene which are expressed in the cells from secondary heart field. All of these three cell populations were positive for FGF10 and dHand (Figure 3B). On the contrary, Tbx5 and eHand genes, which are expressed in the cells from primary heart field [22], were detected only in MSH (Figure 3B). The expression of neural RNA-binding protein Musashi1, marker of neural stem/progenitors [22], was also detected in CSH2 and MSH populations but not in CSH1 cells. In accordance with the recent publications that the heart harbors neural crest-derived multipotent stem/progenitor cells, we analyzed the expression of neural crest-related marker genes AP2α, Pax3, and ErbB3 [23]. Only MSH cells were positive for the three neural crest-related marker genes. Although all cell populations expressed epithelial-mesenchymal transition marker genes Snail and Twist [24], expression of Snail was relatively poor in CSH1 cells.

### Transplantation of Sca-1^+^ cells into the infarcted heart

#### Cardiac function

**Figure 4:**
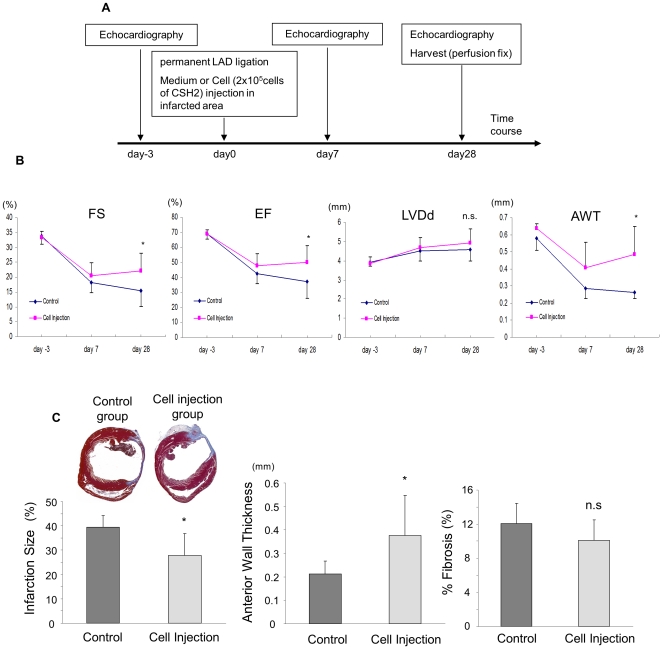
Transplantation effect of cardiac multipotent cells into infarction area of mouse heart. A, Schematic representation of the time course of in vivo experiment. B, Cardiac function and dimensions analyzed by echocardiography. (*p<0.05 *vs* control). Abbreviations: FS, fractional shortening; EF, ejection fraction; LVDd, left ventricle end-diastolic dimension; AWT, end-diastolic anterior wall thickness. C, Heart function studies at four weeks after LAD ligation and cell transplantation. Left upper panels show representative heart sections after Masson's trichrome staining. Left lower panel shows calculated infarction size. Middle panel shows anterior wall thickness of infarcted area. Right panel shows area of fibrosis (%) calculated by computer software. (*p<0.05 *vs* control).

Based on our *in vitro* data, CSH2 were used for transplantation studies in experimental mouse model of MI (Figure 4A). There was no significant difference between cell injected and control groups in the cardiac function indices (LVFS and LVEF), dimensions of LV during diastole (LVDd), and anterior wall thickness (AWT) at base-line levels (Figure 4B). One week after LAD ligation, both groups showed deterioration of cardiac function, slight dilatation of LV dimensions, and decrease of LV wall thickness. However, 4-weeks after LAD ligation, cell injected group showed significantly improved cardiac function and increased wall thickness in the infarct area as compared to control group. Cell transplantation had no significant effect on LV dilatation after MI.

Four weeks after their respective treatment, significantly attenuated infarct size was observed in the animals treated with cardiac stem cells (Figure 4C). The infarct size was 28% of LV wall in the cell injected group which was significantly smaller as compared to the control animal hearts wherein the infarct size was 40% of LV wall (n = 3/group). On the other hand, the percent fibrosis area calculated with computer software from the Masson-trichrome stained heart tissues showed weak tendency of reduction in the cell transplanted animal hearts, however, reduction was insignificant as compared to control animals (Figure 4C).

#### Neovascularization

**Figure 5:**
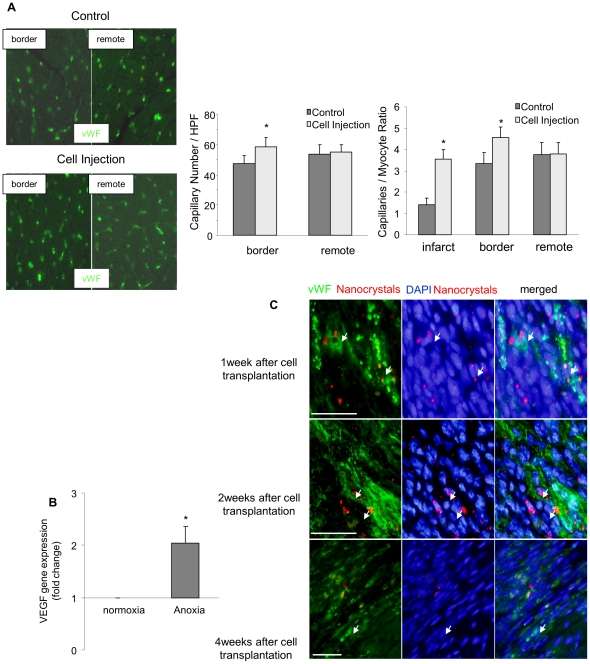
Neovascularization of the infarcted myocardium by cell transplantation. A, Left panels show endothelial cell immunostaining with anti-vWF (green) and DAPI (blue) of control and cell injection group in border zone and remote zone of infarcted heart. Middle panel shows quantitative estimation of capillary density expressed as the number of capillaries per high power magnification field. Right panel shows quantitative estimation of capillary density expressed as capillaries per myocyte. (*p<0.05 *vs* control). B, Relative mRNA expression level of VEGF under normoxic and anoxic conditions analyzed by real-time RT-PCR. (*p<0.05 *vs* control). C, Representative immunostaining of endothelial cells with anti-vWF (green) one, two, and four weeks after transplantation of nanocrystal-labeled cells (red) to detect direct incorporation of transplanted cells into newly formed vessels. Bar = 50 µm.

The number of capillaries and their number distribution per myocyte were counted at high power magnification field. Capillary density was increased in border and infarcted zones of cell transplanted group as compared to control group (Figure 5A) whereas no significant difference in capillary density in the remote zones. To evaluate whether the neovascularization was induced by some paracrine factors from transplanted cells, CSH2 cells were stimulated by hypoxia to mimic the *in vivo* ischemic environment. After 2-hours hypoxia, there was 2-fold increase in VEGF gene expression in CSH2 cells as compared to CSH2 cells cultured under normoxia (Figure 5B). We also observed that 1-week after cell transplantation, nanocrystal (red) labeled transplanted cells were clearly incorporated into vWF^+^ microvessels located between cardiomyofibers (Figure 5C). Two and 4-weeks after cell transplantation, cells positive for both nanocrystals (red) and vWF^+^ (green) were detected in infarcted area.

#### Myogenesis

**Figure 6:**
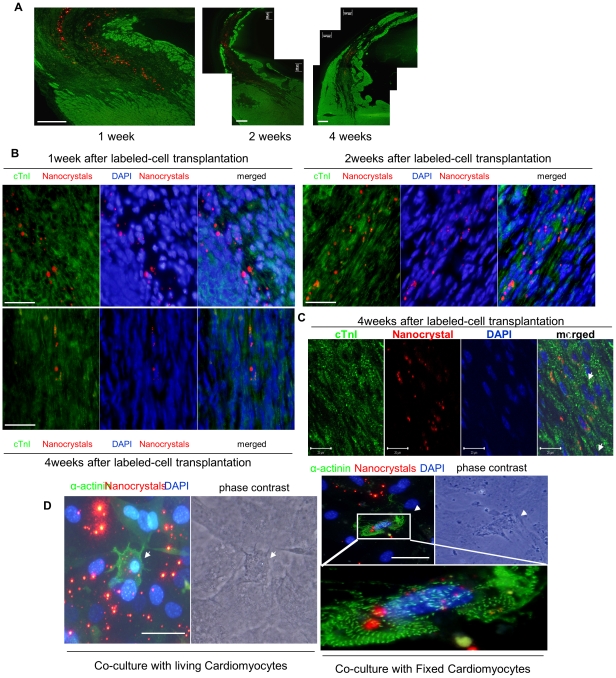
Regeneration of cardiomyocytes by cardiac multipotent cell. A, Heart samples were stained for cardiomyocyte-specific marker cardiac troponin-I (green) one, two, and four weeks after cell transplantation. Transplanted nanocrystal-positive cells (red) accumulated into infarction area and could not detect in remote area at any time point. Bar = 200 µm. B, Representative immunostaining of cTn-I (green) after transplantation of nanocrystal-labeled cells (red) to detect direct regeneration of cardiomyocytes from transplanted cells at one, two, and four weeks after cell transplantation. Bar = 50 µm. C, Representative immunostaining results for cTn-I (green) using confocal microscope four weeks after transplantation of nanocrystal-labeled cells (red). Arrows indicate double positive cells for cTn-I and nanocrystal. Bar = 20 µm. D, Representative immunostaining of α-sarcomeric actinin (green) and DAPI (blue) in co-culture system. White arrows in left panels indicate cardiomyocyte (magnified from the white box) formed from multipotent cardiac cell (double positive for nanocrystal (red) and α-sarcomeric actinin; green) in co-culture system with living cardiomyocytes. Bar = 50 µm. White arrows in right panels indicate double positive cell in co-culture system with PFA-fixed cardiomyocytes. Bar = 50 µm.

We tracked cardiomyogenic fate of the transplanted cells using red fluorescence nanocrystals *in vivo* for up to 4-weeks after transplantation. The transplanted cells were observed only in infarcted area but were not detected in non-infarcted area (Figure 6A). One week after cell transplantation, extensive presence of the transplanted cells was observed at the site of the cell graft which appeared small and round with weak positivity for cTn-I (Figure 6B). Two and 4-weeks later, many nanocrystal-positive cells were aligned parallel to the native microfibers and expressed cTn-I (Figure 6C). These findings indicated slow and progressive morphological transformation of transplanted cells into cardiomyocytes. Despite clear evidence of differentiation potential of CSH2 cells into mature cardiomyocytes *in vivo*, this cell population failed to adopt mature cardiac phenotype *in vitro* even if they expressed cardiomyocyte lineage specific genes and proteins. However, CSH2 cells co-cultured with cardiomyocytes indeed transformed into cardiomyocytes (Figure 6D).

## Discussion

We report successful isolation of Sca-1^+^ cells from adult mice heart based on their functional competence. The isolated Sca-1^+^ cells possessed multiple stem cell-related features and exhibited great potential for myocardial regeneration in a mouse model of acute MI. Gene profiling studies suggested that CSH2 cells originated from precursor cells of secondary heart field and showed functional superiority in terms of proliferation, clonogenicity, self-renewal, and Sca-1 expression besides multiple stem cell-related genes as compared to MSH. Contrarily, MSH cells, isolation of which was based on Sca-1 expression alone, were heterogeneous and contained cell populations from different sources including primary heart field, secondary heart field, and neural crest-derived cells.

Purification of a cardiac stem cell population from the heart is challenging, especially due to lack of specific surface markers. We therefore exploited clonogenic potential of the stem cells, which is considered as one of the functional feature of stem/progenitor cells [25], in combination with Sca-1 expression for their purification. We cultured the heart-derived cells in serum-free culture conditions at a very low density which alleviated their cross-contamination from the presence of mature cells in the culture besides recovery of sub-clonal cell populations with strong survivability and proliferation. Interestingly, some of these cell populations thus obtained showed spontaneous high Sca-1 positivity with very low c-kit expression stem/progenitor cell marker [1], [8]. This may be attributed to the difference in the culture conditions and different expression period of these two surface markers during maturation of heart [26]. CSCs in their undifferentiated status express stem-cell antigens c-kit, MDR1, and Sca-1 either alone or in combination, however only a small percentage of cells is positive for anyone of the three antigens alone [27]. Of these three populations, ckit^+^ CSCs are the most extensively studied and well characterized CSCs for stemness characteristics including clonogenicity, self renewal and multipotentiality [28]. On the contrary, Sca-1^+^ progenitor cells constitute a predominant stem cell population in the heart as compared to the c-kit^+^ cells [1].

We observed that the purified Sca-1^+^ cells expressed mature cardiomyocyte gene markers including β-MHC and MLC2α. Although α-MHC is predominant isoform of MHC in adult mouse heart, β-MHC isoform is strongly expressed in both atrial and ventricular muscle in the developing heart [26]. Additionally, MLC2α is expressed in myocardial cells during early stage of cardiac development as one of the earliest marker for differentiated cardiomyocytes [29]. Therefore, the gene expression of β-MHC and MLC2α markers clearly represented early commitment to cardiomyogenic lineage.

The purified Sca-1^+^ cells also expressed Isl-1 gene, a marker for secondary heart field [10], [22]. Isl-1^+^ cells are considered as cardiovascular precursors with the ability to differentiate into the three main cardiac cell lineages [9] and post-natal mouse heart [5]. Brachyury (Bry)^+^/Flk-1^+^ cells from mouse ES cell derived embryoid bodies differentiated into three cardiac cell lineages [30]. Similarly, cardiac progenitor cells from developing mouse embryo differentiated into smooth muscle cells and cardiomyocytes [26]. Although the definite hierarchy of these reported cardiac precursor/progenitor cells remains unclear, it is suggested that Bry^+^/Flk-1^+^ cells may precede the separation of Isl-1^+^ cells and, the Nkx2.5 expressing progenitor cells may be downstream of the Bry^+^/Flk-1^+^ cells and Isl-1^+^ cells because of their bi-potential differentiation capacity [25]. In accordance with our data, CSH2 cell population is positioned between Isl-1^+^ and Nkx2.5 due to expression of Isl-1 and Flk-1, and lack of Nkx2.5 during the undifferentiated state while these cells expressed Nkx2.5 after differentiation respectively.

Our *in vivo* studies in the experimental animal model provided a clear evidence of differentiation potential of CSH2 cells into mature cardiomyocytes. We believe that rate of cardiomyogenic differentiation and the improvement in functional indices of the heart would have been better if the cells were transplanted during chronic phase after LAD ligation when the acute phase inflammatory responses had subsided. Contrary to the *in vivo* data, these cells showed low incidence of differentiation to adopt mature cardiac phenotype *in vitro* which may be attributed to lack of required multiple cues for maturation [31]. A recent report suggests that a direct contact of cardiac stem/progenitor cells with cardiomyocytes is a pre-requisite and critical for their differentiation [5]. These cells co-cultured with cardiomyocytes were indeed transformed into myocytes (Figure 6D). Although we only considered α-sarcomeric actinin positive cell with single nucleus as the differentiated cell, we could not rule out the possibility that these α-sarcomeric actinin positive cells were formed by fusion with co-cultured cardiomyocytes. In order to exclude the possibility of cell fusion between multipotent cardiac stem/progenitor cells and living cardiomyocytes, we co-cultured cardiac stem/progenitor cells with paraformaldehyde-fixed neonatal rat cardiomyocytes with cardiomyocytes conditioned medium [5]. These results showed that both secreted factors in the cardiomyocyte conditioned medium and the membrane bound factors in the cardiomyocytes were essential for induction of cardiomyogenic differentiation of multipotent stem cells. One week after co-culture, small number (similar to co-culture with living cardiomyocyte) of CSH2 cell population stained positively for α-sarcomeric actinin (Figure 6D). These data suggested that CSH2 cell population really harbored true immature cell population which differentiated into cardiomyocytes under optimal conditions.

Despite promising data, the study has some limitations. Although the cells showed cardiomyogenic regeneration capacity and therapeutic effects post-engraftment, the frequency of cardiomyogenic differentiation of CSH2 cells *in vitro* conditions was lower than our anticipation. Although the underlying reason is unclear, a recently published study using four cardiac progenitor cell types including Isl-1^+^/Nkx2.5^−^, Isl-1^−^/Nkx2.5^+^, Isl-1^+^/Nkx2.5^+^, and Isl-1^−^/Nkx2.5^−^ cells to compare their differentiation capacity into cardiomyocytes and smooth muscle cells under two different culture conditions [32]. Interestingly, only Isl-1^+^/Nkx2.5^−^ cardiac progenitor cells showed significant increase in cardiomyogenic differentiation under specific culture conditions. The authors suggested that the cardiomyogenic potential of Isl-1^+^/Nkx2.5^−^ was modulated by microenviromental geometric cues. These observations supported our results that CSH2 with Isl-1^+^/Nkx2.5^−^ cells represented a true population of cardiac progenitor cells. Secondly, inspite of immunohistological evidence that about cardiomyogenic differentiation, future studies would be required showing functional integration of the differentiated cells. Lastly, a direct comparison of the clonally selected Sca-1^+^ cells with MACS sorted Sca-1^+^ cells would further show the significance of our new method of Sca-1+ cell purification.

In conclusion, we have demonstrated that adult mouse heart harbors Sca-1^+^/Isl-1^+^/Nkx2.5^−^ cells with their unique functional features, clonality and self-renewal capacity. This cell population originated from cells of secondary heart field and showed multilineage differentiation potential. Transplantation of these cells into infarcted heart significantly preserved the heart function and attenuated infarction size expansion. Further investigation into the potential of adult heart-derived Sca-1^+^/Isl-1^+^/Nkx2.5^−^ stem/progenitor cells would be required to utilize these promising candidates for the heart cell therapy.

## Supporting Information

Figure S1 **Differentiation potential of isolated Sca-1 high or low expressing cell populations into smooth muscle cell lineage.** A, All cell populations were stained for smooth muscle cell specific marker calponin (green) and DAPI (blue) both before induction (Control) and after induction (Induction). Magnification = ×400. B, Calponin positive cell number was calculated in all cell populations both before induction (Control) and after induction (Induction). The positive rate was presented as the ratio of calponin positive cell number to total cell number (*p<0.01 *vs* Control; **p<0.05 *vs* Control; †*p*<0.01 *vs* all other cell populations; ‡*p*<0.01 *vs* CSH1, CSH2, and CSL1; §*p*<0.01 *vs* CSH2 and CSL1). C, Expression of smooth muscle cell specific genes analyzed by RT-PCR. RNA extracted from whole heart of adult mouse (mH) was used as positive control.
(TIF)

Figure S2 **Differentiation potential of isolated Sca-1 high or low expressing cell populations into endothelial cell lineage.** A, All cell populations were stained for endothelial cell specific marker von Willebrand Factor (green) and DAPI (blue) after induction. Magnification = ×400. B, vWF positive cell number was calculated before induction (Control) and after induction (Induction). The positive rate was presented as the ratio of vWF positive cells to total cell number (*p<0.01 *vs* Control, †*p*<0.01 *vs* all other cell populations). C, Expression of endothelial cell specific genes analyzed by RT-PCR. RNA extracted from whole heart of adult mouse (mH) was used as positive control. Arrow indicated the expression of endothelial cell specific genes after induction.
(TIF)

Text S1 **Supporting Information.**
(DOC)
