# Supplementary figures and images for: Identification and Characterization of a Novel Multipotent Sub-Population of Sca-1+ Cardiac Progenitor Cells for Myocardial Regeneration
Source: PLoS One. 2011 Sep 28;6(9):e25265. doi: 10.1371/journal.pone.0025265 (PMC3182214; doi:10.1371/journal.pone.0025265)

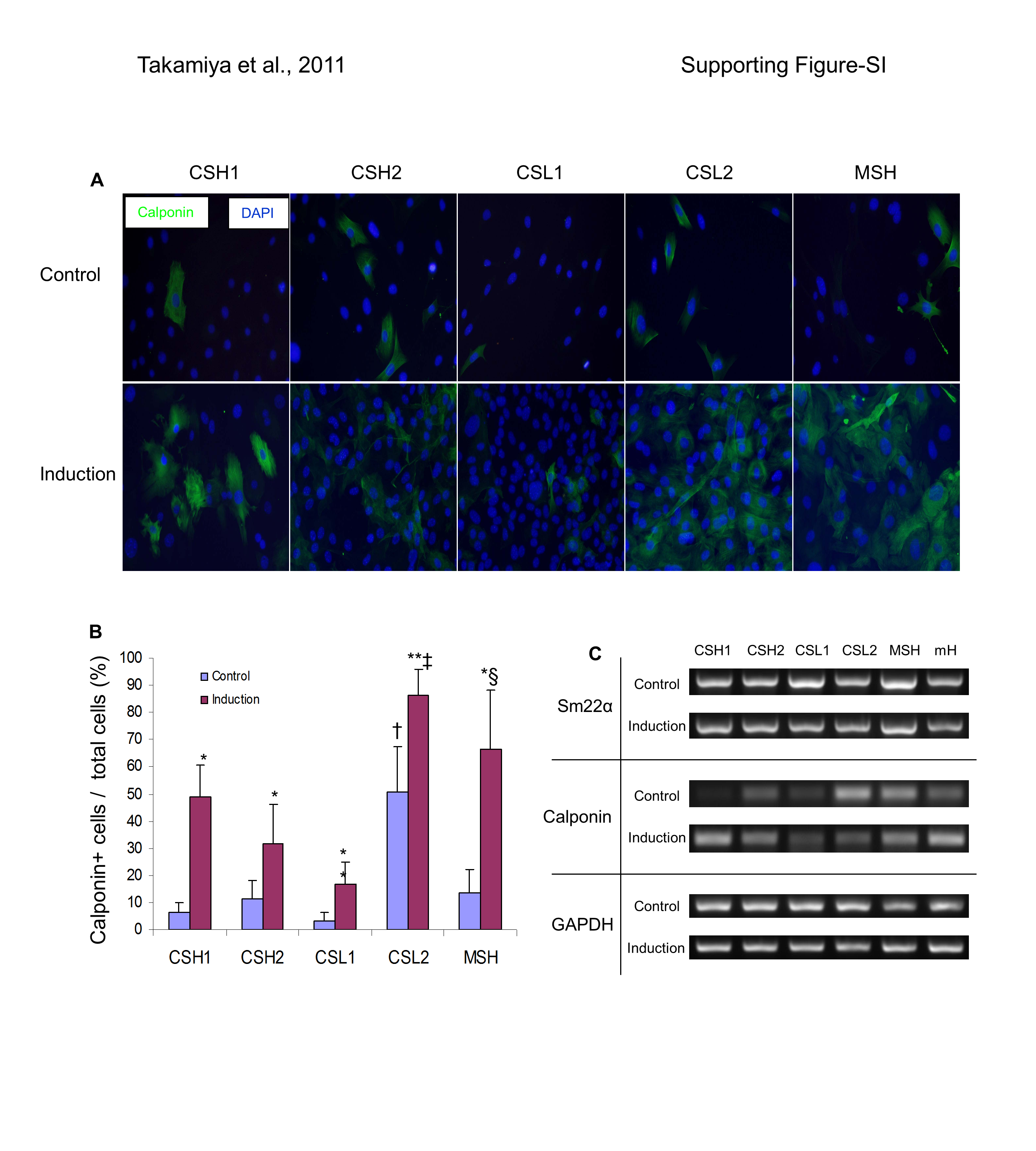

Supplement: Figure S1 — Differentiation potential of isolated Sca-1 high or low expressing cell populations into smooth muscle cell lineage. A, All cell populations were stained for smooth muscle cell specific marker calponin (green) and DAPI (blue) both before induction (Control) and after induction (Induction). Magnification = ×400. B, Calponin positive cell number was calculated in all cell populations both before induction (Control) and after induction (Induction). The positive rate was presented as the ratio of calponin positive cell number to total cell number (*p<0.01 vs Control; **p<0.05 vs Control; †p<0.01 vs all other cell populations; ‡p<0.01 vs CSH1, CSH2, and CSL1; §p<0.01 vs CSH2 and CSL1). C, Expression of smooth muscle cell specific genes analyzed by RT-PCR. RNA extracted from whole heart of adult mouse (mH) was used as positive control. (TIF) [file pone.0025265.s001.tif]

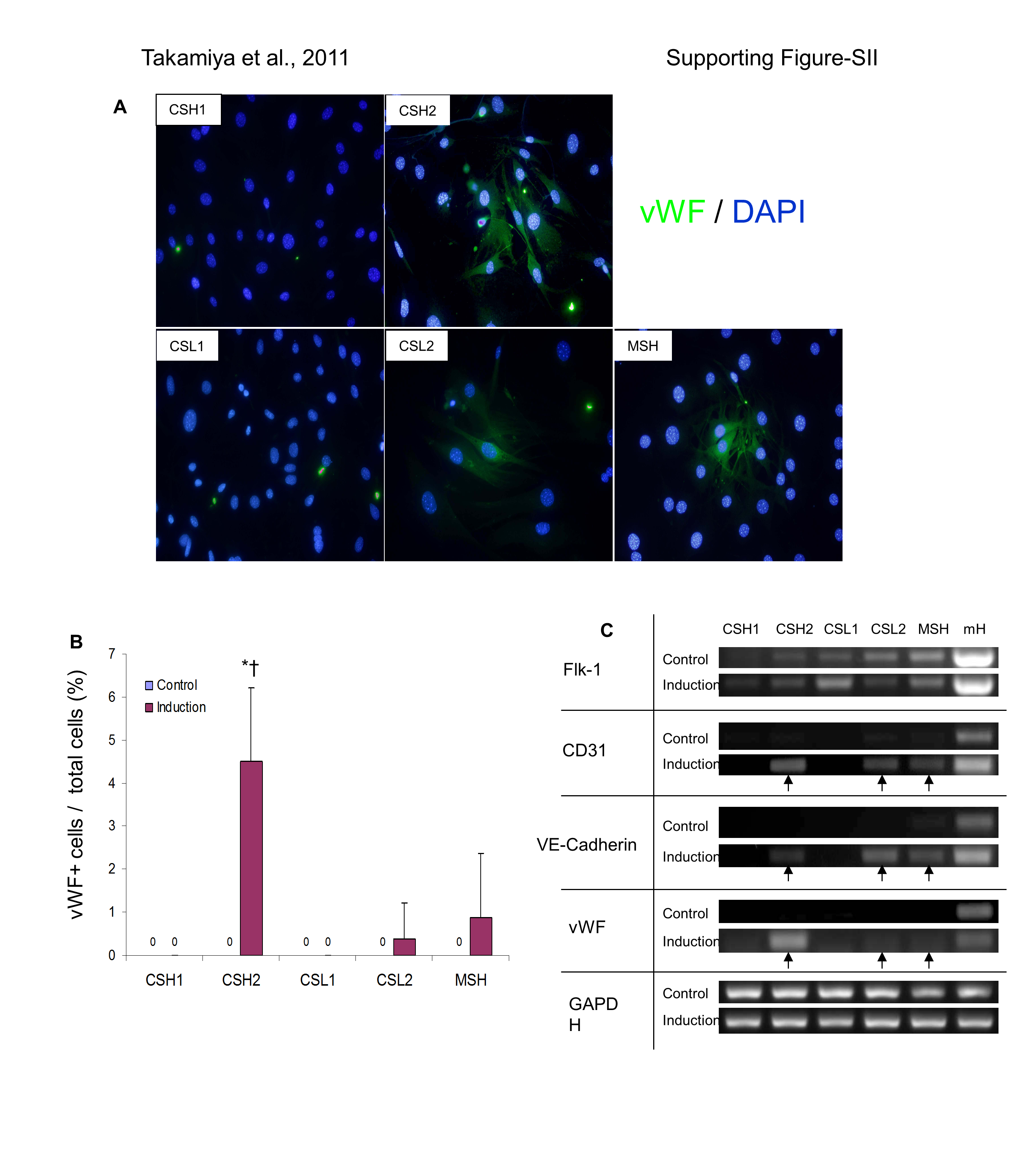

Supplement: Figure S2 — Differentiation potential of isolated Sca-1 high or low expressing cell populations into endothelial cell lineage. A, All cell populations were stained for endothelial cell specific marker von Willebrand Factor (green) and DAPI (blue) after induction. Magnification = ×400. B, vWF positive cell number was calculated before induction (Control) and after induction (Induction). The positive rate was presented as the ratio of vWF positive cells to total cell number (*p<0.01 vs Control, †p<0.01 vs all other cell populations). C, Expression of endothelial cell specific genes analyzed by RT-PCR. RNA extracted from whole heart of adult mouse (mH) was used as positive control. Arrow indicated the expression of endothelial cell specific genes after induction. (TIF) [file pone.0025265.s002.tif]
