# Supplementary material for: Identification and Characterization of a Novel Multipotent Sub-Population of Sca-1+ Cardiac Progenitor Cells for Myocardial Regeneration
Source: PLoS One. 2011 Sep 28;6(9):e25265. doi: 10.1371/journal.pone.0025265 (PMC3182214; doi:10.1371/journal.pone.0025265)
Supplement: Text S1 — Supporting Information. (DOC) [file pone.0025265.s003.doc]

**Supporting Information**

**Text S1**

**Flow cytometry**

The cells were detached using 1x trypsin/EDTA solution (Invitrogen) and washed wash x3 with FBS containing cell culture medium. The cells were counted and divided into triplicate samples (1x10^5^ cells/ sample). For immunostaining, the cells were suspended in 50-100µl of FACS buffer (PBS containing 0.5%BSA and 2mM EDTA) and incubated with primary antibody conjugated with FITC fluorescence marker for 30 minutes at 4°C. The cells were later washed x2 with FACS buffer and re-suspended in 1 ml FACS buffer and passed through 70µm-filter before analysis. The antibodies used during FACS analysis are shown in Table-S1.

**Differentiation assay**

These cells were cultured in specific differentiation mediums for 2 weeks. For smooth muscle cell induction, cells were cultured with DMEM/F12 supplemented with 10%FBS and 50ng/ml of PDGF-BB (Peprotech). For endothelial cell induction, cells were cultured with EBM-2 medium (Lonza) supplemented with EGM-2 SingleQuots kit (Lonza) except hydrocortisone on fibronectin-coated dish. For cardiomyocyte induction, cells were cultured with DMEM/F12 supplemented with 2%FBS, B27 supplement, 100ng/ml of BMP-2 (Prospec), and 100ng/ml of FGF-4 (Prospec) on gelatin-coated dish. For quantitative analysis, these cardiogenic differentiation assays were performed on 2-well chamber slides and stained with specific antibodies. All cell populations before induction and after induction with individual differentiation medium were fixed with 4%PFA and stained for anti calponin antibody (Abcam), anti von Willebrand Factor (vWF) antibody (Chemicon), or anti cardiac-myosin heavy chain (cMHC) antibody (Abcam). All cells were subsequently incubated with secondary antibody conjugated to Alexa Fluor 488 (Invitrogen). Nuclei were stained with DAPI. Five high magnification fields were selected randomly from all wells and positive and total number of cells was counted. Staining rate was calculated as the ratio of positive cell number to total cell number and the average from five different fields were presented.

To evaluate the differentiation potential into mesenchymal cell lineage, Sca-1 high expressing cell populations were differentiated into osteogenic, chondrogenic, and adipogenic cell lineage using StemPro Osteogenesis Differentiation Kit, StemPro Chondrogenesis Differentiation Kit, and StemPro Adipogenesis Differentiation Kit (all from Invitrogen) respectively according to manufacture’s instruction. Osteogenic differentiation was detected by von Kossa staining [14]. Chondrogenic differentiation was detected by Toluidine Blue O staining [14]. Adipogenic differentiation was detected by Oil Red O staining [14].

**Sphere formation assay**

All cell populations were seeded in the 60 mm ultra-low attachment dish (Corning) at a density of 2x10^3^ cells/cm^2^ with low-serum expansion medium. Two weeks after initial seeding, the sphere-like cell clusters were fixed with 4% paraformaldehyde (PFA) and stained for Ki67 antigen expression using Ki67 specific antibody (Abcam; 1:1000 dilution). The primary antigen-antibody reaction was detected using Alexa Fluor 488 conjugated secondary antibody (Invitrogen). The nuclei were visualized by staining with 4', 6-diamidino-2-phenylindole (DAPI).

**Myocardial infarction model and cell transplantation**

Experimental myocardial infarction was developed in young female C57BL6 mice (12 weeks old; 20-25 g body weight). The animals were anesthetized by intraperitoneal injection of Ketamine/Xylazine (2.5 mg and 0.5 mg respectively). After tracheal intubation with 22-gauge intravenous catheter (Exelint International), the respiration of mice was controlled artificially by mechanical ventilator (Harvard Apparatus). Normal chest expansion was noted similar to a conscious mouse. The mouse myocardial infarction model was produced as previously described with minor modifications [15]. Briefly, the heart was exposed by left thoracotomy and left auricle was gently retracted to expose the left anterior descending (LAD) coronary artery which was ligated with 6-0 silk under the microscope. Ten minutes after LAD ligation, 20µl basal DMEM/F12 medium without cells (control group) or containing 2x10^5^ cells (Cell Injection group) were injected into infarction and border zones through 29-gauge needles. After cell injection, the chest was sutured with 6-0 silk and all mice were allowed to recover.

**Morphological analysis**

At the end of experimental period, the animals were killed and the hearts were perfusion-fixed with 4% PFA, cut transversely, embedded in paraffin, and cut transversely into 6-8µm thick sections at the level of papillary muscle. The tissues sections were stained with haematoxylin-eosin and Masson’s trichrome and were scanned. The infarction size, anterior wall thickness, and percent fibrosis were measured on the scanned images of the tissue sections stained with Masson’s trichrome using ImageJ software (NIH). The ratio of scar length to left ventricular circumferences of the endocardium and epicardium was determined and expressed as a percentage of infarct size (% of LV) which was calculated as (infarct area/total LV area)×100 [16]. Anterior wall thickness was measured at three different points in infarction area of anterior wall perpendicularly and average was taken. Transverse fibrosis and total left ventricular areas were measured and expressed as percent fibrosis [16].

**Echocardiography**

The mouse heart function was assessed by transthoracic echocardiography before surgery (baseline), one week and four weeks after surgery using 7-15 MHz probe on iE33 Echocardiography system (Philips). The animals were sedated and the hearts were imaged in 2D and M-mode from parasternal long axis view, and all measurements were obtained at the level of the largest left ventricular (LV) diameter. Anterior and posterior end-diastolic wall thickness and LV end-diastolic dimension (LVDd) and LV end-systolic dimension (LVDs) were measured from M-mode view at the papillary muscle level. LV fractional shortening (LVFS) and ejection fraction (LVEF) were calculated using LVFS= (LVDd-LVDs)/LVDd x100 and LVEF= [(LVDd^3^-LVDs^3^)/LVDd^3^] x100 respectively and the results were expressed as percentage.

**Capillary density analysis**

Transverse sections (6µm) were obtained from 4% PFA fixed heart tissue and incubated with anti vWF antibody (Chemicon) followed by incubation with secondary antibody conjugated to Alexa Fluor 488 (Invitrogen). Nuclei were stained with DAPI. Capillary density was determined at randomly selected high magnification fields in both border zone and intact remote zone and the average were considered as capillary density. The number of capillaries surrounding one myofiber was calculated from randomly selected five myofibers at two different high magnification fields in infarct area and remote zone and averaged. The capillary density was calculated as the number of capillaries surrounding one myofiber [17].

**Table-S1:** Antibodies used for FACS analysis

Antibody specificity Source Cat# dilution used

anti-Sca-1 Militenyi Biotech 130-092-529 1:10

anti-mouse CD34 Beckman Coulter 732344 1:20

anti-mouse CD45 Beckman Coulter 732147 1:20

anti-mouse CD31 Beckman Coulter 733290 1:10

anti-mouse CD29 eBioscience 12-0291 1:20

anti-mouse CD117 (ckit) BD Pharmingen 553355 1:20
